# Morita Therapy for Persistent Postural-Perceptual Dizziness (PPPD): A Case Report

**DOI:** 10.7759/cureus.84263

**Published:** 2025-05-16

**Authors:** Shogo Saito, Fumiyuki Goto

**Affiliations:** 1 Department of Otolaryngology, Goto Otolaryngology Clinic, Machida, JPN; 2 Department of Otolaryngology, Tokai University School of Medicine, Isehara, JPN

**Keywords:** case report, chronic dizziness, morita therapy, persistent postural-perceptual dizziness, psychotherapy

## Abstract

Persistent postural-perceptual dizziness (PPPD) is a chronic functional vestibular disorder that persists for more than three months and significantly impairs patients' mental health and quality of life (QoL). The onset and persistence of PPPD are believed to involve excessive attention to symptoms, requiring approaches from both physical and psychological perspectives. Morita therapy (MT) is a psychotherapy that aims to improve symptoms through defocusing attention from symptoms. This article reports a case in which five sessions of MT were conducted for a PPPD patient who had shown resistance to conventional treatments such as pharmacotherapy with selective serotonin reuptake inhibitors (SSRIs) and vestibular rehabilitation (VR). As a result of the intervention, the patient's excessive attention to dizziness symptoms decreased and participation in previously avoided activities was promoted, leading to marked improvements in dizziness symptoms, anxiety, depressive symptoms, and QoL. These effects were maintained at the three-month follow-up after treatment completion. This case suggests that MT could be a promising treatment option for PPPD patients who do not respond to existing treatments.

## Introduction

Persistent postural-perceptual dizziness (PPPD) is a chronic functional vestibular disorder characterized by symptoms of dizziness, unsteadiness, or non-spinning vertigo that persist for more than three months [1]. It is marked by the worsening of symptoms in response to specific postures, motions, and visual stimuli. Although the pathophysiology of PPPD differs from that of other vestibular and psychiatric disorders, psychological factors such as anxiety are thought to amplify its symptoms [2]. Furthermore, chronic dizziness causes psychological distress and activity limitations, significantly reducing quality of life (QoL) [3]. Spontaneous remission is rare, and without treatment, dizziness typically persists [4], highlighting the need for interventions addressing both physical and psychological aspects.

Although treatments such as selective serotonin reuptake inhibitors (SSRIs), vestibular rehabilitation (VR), and cognitive behavioral therapy (CBT) have shown promise for PPPD [2], a standardized treatment protocol has yet to be established. It has been suggested that increased body vigilance contributes to the onset of PPPD [5]. Given that chronic dizziness frequently co-occurs with or can lead to somatic symptom disorder [6], excessive attention to physical sensations is thought to contribute to both the onset and persistence of PPPD. Based on our prior experience observing improvement in phobic postural vertigo (PPV), a related condition, with autogenic training (AT) [7], and reports of the effectiveness of acceptance and commitment therapy (ACT) for PPPD [8], we considered that psychotherapies aimed at reducing symptom preoccupation (not limited to CBT) might be beneficial for PPPD.

Developed by Japanese psychiatrist Shoma Morita in 1919 [9,10], Morita therapy (MT) aims to reduce preoccupation with symptoms through acceptance of symptoms and activation of behavior. Historically, MT was primarily known as an inpatient treatment for anxiety disorders. In this traditional approach, patients learned to accept their symptoms and engage in constructive behaviors by undertaking daily life tasks within a structured environment, regardless of their symptoms. In contemporary practice, MT has largely evolved into an outpatient, dialogue-centered approach. Moreover, its application has expanded to a broader range of conditions where psychological factors play a significant role. These include mood disorders and somatic symptom disorders, as well as conditions like PPPD, where physical complaints are prominent and often intertwined with psychological distress.

Herein, we present a case of PPPD, previously unresponsive to standard therapies, in which the addition of MT to conventional treatment resulted in marked symptom improvement.

## Case presentation

Patient information

The patient, a 36-year-old Japanese woman living with her husband and working as a full-time homemaker, presented seeking relief from persistent dizziness and associated distress.

History of present illness

The patient developed persistent dizziness following dental treatment. An initial evaluation by an otolaryngologist revealed mild vestibular dysfunction, deemed insufficient to fully explain her symptoms. Neurological assessment found no brain abnormalities. VR was attempted for eight months but proved ineffective. Due to co-occurring headaches, anxiety, and depressive symptoms, she consulted a psychiatrist. While treatment with the SSRI fluvoxamine (50 mg) for six months alleviated her depressive symptoms, the dizziness persisted. Consequently, the patient sought specialized care at our hospital. Her dizziness was exacerbated by upright posture, active or passive motion, and exposure to moving or complex visual stimuli. Based on established diagnostic criteria [1], she was diagnosed with PPPD by the attending physician. At our hospital, her treatment with fluvoxamine (50 mg) was continued, and she received instruction in vestibulo-ocular reflex (VOR) and vestibulo-spinal reflex (VSR) exercises, based on the VR guidelines from the Japan Society for Equilibrium Research [11]. Additionally, given her lack of response to conventional treatments and the suspected role of psychosocial factors in maintaining her symptoms, psychotherapy was recommended and initiated.

Assessment

Audio-vestibular examinations conducted at our hospital, including pure tone audiometry, video head impulse test (vHIT), cervical vestibular evoked myogenic potentials (cVEMP), ocular vestibular evoked myogenic potentials (oVEMP), and posturography, revealed no significant abnormalities. At baseline psychological evaluation, the Dizziness Handicap Inventory (DHI) [12] was used to assess handicap due to dizziness, and the Niigata PPPD Questionnaire (NPQ) [13] was used to assess the severity of PPPD symptoms. Her DHI score was 46, indicating a severe dizziness-related handicap [14], and her NPQ score was 28 (a score ≥27 suggests PPPD-related symptoms). The Hospital Anxiety and Depression Scale (HADS) [15] was used to assess psychiatric symptoms. HADS is designed to detect anxiety and depression and assess their severity in patients with physical symptoms. Her HADS scores were 9 for anxiety and 9 for depression. These scores are in the borderline range (8-10), suggesting possible anxiety and depressive symptoms.

Therapeutic intervention

The patient underwent five sessions of MT with a clinical psychologist specializing in MT (seven years of training). These sessions, each lasting 50 minutes, were conducted over a total period of five months. Initially, sessions were held biweekly, with subsequent intervals adjusted from two to six weeks according to the patient's progress. The patient completed the DHI, NPQ, and HADS questionnaires before each session to monitor progress. Key themes and interventions for each session are summarized in Table 1.

**Table 1:** Summary of Morita therapy sessions

Session	Focus	Session outline	Patient's perspective
#1	Psychoeducation	Provided psychoeducation based on Morita Therapy regarding the vicious cycle where attention worsens symptoms. Shared the approach aiming for quality of life (QoL) improvement by accepting dizziness, rather than trying to eliminate it completely.	She recognized she was preoccupied by dizziness and agreed with the therapeutic approach focused on improving QoL.
#2	Identifying "Desire for Life" and promoting activity	Identified the patient's "Desire for Life" (desired goals and activities) and encouraged activities aligned with it.	She reported attending an event of interest, describing this participation as reflecting her 'Desire for Life' and contributing to her QoL improvement.
#3–4	Reviewing activities and deepening acceptance	Reviewed experiences in various daily situations and deepened acceptance-based coping with symptoms and emotions.	She reported successfully taking a trip and resumed dental treatment previously postponed due to dizziness. Although dizziness temporarily worsened, she managed this through acceptance.
#5	Session review	Reviewed the overall therapy sessions and discussed relapse prevention.	She reported days without being conscious of dizziness. She became able to flexibly adjust her activities to improve her QoL and developed acceptance of the dizziness.

Course of therapy

Initially, the patient believed that dizziness was severely disrupting her life and was fixated on its complete elimination. She was constantly preoccupied with dizziness and experienced significant anxiety. She repeatedly engaged in behaviors aimed at eliminating the dizziness, which led to exhaustion, and began avoiding previously enjoyed activities such as social events and travel due to fear of symptom exacerbation.

Session 1

Psychological education based on MT principles was provided. Key concepts included: (1) Negative emotions (like anxiety) and positive desires are interconnected, with anxiety often intensifying due to a strong underlying "Desire for Life" (e.g., the desire for health and activity). (2) The "mechanism of toraware" (mental preoccupation) was explained as a vicious cycle where efforts to eliminate sensations paradoxically increase focus on them, thereby amplifying them. MT posits that the "Desire for Life" has two aspects. First, a narrow focus of this desire on symptom elimination paradoxically intensifies toraware and maintains the vicious cycle. Second, directing this desire toward broadly enriching life can divert attention from symptoms, thereby helping to break the vicious cycle. Based on this understanding, the therapeutic direction shifted from complete symptom elimination to accepting the dizziness and focusing the "Desire for Life" on improving QoL to reduce associated suffering, an approach the patient accepted.

Session 2

The patient reported attending a neighborhood event. Although anxiety and fatigue temporarily worsened her dizziness, she managed by leaving early and still enjoyed the experience. The therapist highlighted that adjusting activities based on her condition, rather than striving for symptom elimination, facilitated enjoyment. The therapist framed her desire for events and travel as a reflection of her "Desire for Life," establishing a treatment goal to act toward fulfilling this desire while accepting the accompanying anxiety.

Sessions 3 and 4

The patient reported successfully taking a trip. She described managing episodes of increased dizziness due to fatigue by resting calmly, which allowed her to enjoy the trip without significant anxiety. The therapist acknowledged and supported her growing ability to accept and manage her physical sensations and emotions. Additionally, she resumed dental treatment, which had been postponed due to dizziness. While this briefly increased her focus on dizziness, it did not impede her daily activities.

Session 5

The patient reported experiencing days without being conscious of dizziness. Following the principles of MT, therapeutic progress was reviewed across symptom improvement, behavioral change, and self-acceptance [9]. Regarding behavior, she stated, "Previously, I never considered stopping my plans midway even when I felt unwell. Now I can think that it's okay to stop if it's not working out, and I've learned to take better care of my body." Regarding self-acceptance, she reflected, "Before, I was afraid of dizziness and tried to avoid it, which intensified my fear and made me unable to act. Even now, I become anxious when dizziness intensifies, but I've come to accept that I have to accept it. Rather than focusing on preventing dizziness and anxiety from becoming severe, I prioritize taking action and enjoying activities."

**Figure 1:**
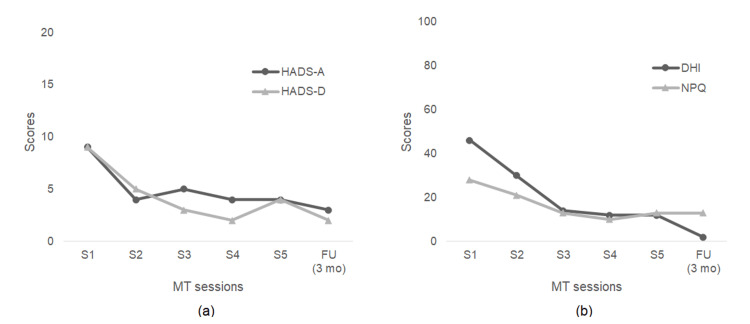
Changes in DHI, NPQ, and HADS scores over time (a) Changes in HADS-A and HADS-D scores (b) Changes in DHI and NPQ scores DHI: Dizziness Handicap Inventory, FU: follow-up, HADS-A: Hospital Anxiety and Depression Scale–Anxiety subscale, HADS-D: Hospital Anxiety and Depression Scale–Depression subscale, MT: Morita therapy, NPQ: Niigata PPPD Questionnaire.

Over the five sessions, as shown in Figure 1, psychological evaluation scores decreased as follows: DHI from 46 to 12, NPQ from 28 to 13, HADS-A from 9 to 4, and HADS-D from 9 to 4. Treatment was concluded due to marked improvement.

Follow-up (Three Months)

A follow-up session confirmed sustained improvement. While some dizziness persisted, the patient reported accepting the symptoms and functioning well. She reflected on the treatment: "Previously, I was paying too much attention to my body and anxiety. Even now, when I focus on it, the dizziness is there, but usually I don't worry about whether I have dizziness or not. I've come to understand that I can rest when dizziness occurs."

Psychological evaluation scores remained low: DHI 2, NPQ 13, HADS-A 3, HADS-D 2 (Figure 1). The NPQ score remained at 13 at the three-month follow-up, unchanged from the score after five sessions, which may suggest the persistence of some symptoms of PPPD. However, given the significant improvement in the DHI score and her self-reports, these residual symptoms were interpreted as no longer substantially impacting her QoL. This outcome is considered to reflect an increased acceptance of her symptoms and a resolution of toraware (mental preoccupation).

## Discussion

Studies have shown that increased vigilance toward bodily sensations is involved in the development of PPPD [5]. Given the role of attentional preoccupation in PPPD and this patient's presentation, MT, a psychotherapy aimed at reducing such preoccupation through acceptance and behavioral activation, was implemented. The results of this case suggest that MT may be beneficial in improving dizziness symptoms and enhancing QoL in patients with PPPD.

While the mechanism of PPPD onset has not been fully elucidated, a proposed pathophysiology has been described [16]. Following an acute vestibular event or balance disorder, the weighting of vestibular, visual, and somatosensory inputs for balance perception changes. A postural control strategy that relies more heavily on visual and somatosensory inputs is adopted to compensate for impaired vestibular sensation. Normally, this strategy becomes unnecessary as the initial disorder resolves and usual function returns. However, in PPPD, this adaptive postural control system persists long term. As a result, hypervigilance for dizziness-related sensations develops, and even minor stimuli can trigger dizziness. Anxiety about dizziness and falling further maintains this maladaptive postural control system, creating a vicious cycle that perpetuates dizziness. Therefore, interventions targeting this cycle are necessary for improving PPPD symptoms.

Furthermore, chronic dizziness causes psychological distress and activity limitations, significantly reducing QoL [3]. As observed in this case, patients with PPPD may experience a diminished sense of identity when dizziness impairs their daily life and prevents participation in valued activities [17]. Consequently, addressing not only symptom reduction but also the improvement of functional impairment is an important challenge in PPPD treatment.

MT conceptualizes symptom maintenance through a vicious cycle model, which resonates with the proposed pathophysiology of PPPD. MT's model involves mental preoccupation (toraware, a term used in MT literature), understood as resulting from two interacting cycles [10]. One cycle involves an attention-mediated sensory aggravation effect, where focusing attention on specific sensations amplifies them. The other involves ideational contradictions, where attempts to willfully control sensations paradoxically direct more attention toward them, reinforcing the cycle. MT aims to break these vicious cycles by fostering acceptance of symptoms and redirecting focus toward constructive behavior. Additionally, it seeks to improve QoL by channeling energy, previously consumed by efforts to eliminate symptoms, toward realizing the patient's "Desire for Life." Thus, MT's understanding of symptom mechanisms and its treatment goals appear well-aligned with the challenges presented by PPPD. Accordingly, as demonstrated in this case, the intervention was conducted according to the principles of MT as follows [10]: First, explanation and mutual understanding of the "mechanism of toraware" between the therapist and patient led to the patient's insight that attempting to eliminate symptoms contributed to their maintenance. Consequently, the therapeutic goal was established as improving QoL. To enhance QoL, specific actions reflecting the patient's "Desire for Life" were identified, and she was encouraged to engage in them. These experiences facilitated acceptance of symptoms and negative emotions, successfully breaking the vicious cycle.

As reported in this case, implementing MT for PPPD suggested improvements in subjective dizziness symptoms and QoL, as well as reductions in anxiety and depression. MT may be effective for PPPD through mechanisms different from those of other psychotherapies. While CBT generally aims to improve symptoms through cognitive modification, considering the pathophysiological hypothesis of PPPD [16], interventions targeting cognitions that attempt to control symptoms might theoretically enhance attention to symptoms and maintain the vicious cycle. Additionally, ACT shares commonalities with MT in terms of (a) accepting symptoms and (b) acting toward values, aiming to defocus from symptoms [18]. However, while ACT uses exercises to address these two aspects separately, MT promotes symptom acceptance through experiences of being able to act despite symptoms while pursuing life's desires [10]. In other words, MT's distinctive feature is that the practice of actions based on life's desires is inseparably linked to the process of experiential acceptance of symptoms, enabling simultaneous progression of processes that ACT addresses separately. Furthermore, while both AT and MT aim for symptom acceptance, a key difference is that AT primarily seeks physiological stabilization through relaxation and self-hypnosis. In contrast, MT experientially promotes coexistence with symptoms through daily life actions. Therefore, MT may offer new insights for PPPD patients who have not responded to conventional treatments.

Finally, several study limitations warrant consideration. First, since the patient was concurrently receiving SSRIs and VR treatment, it is difficult to definitively attribute the observed symptom improvement solely to MT. However, given the rarity of spontaneous remission in chronic dizziness [4] and the reported limited efficacy of SSRIs and VR in this case, MT likely contributed significantly to the patient's improvement. Second, as a single case study, the results require cautious interpretation, and generalizability is limited. Therefore, the influence of a placebo effect cannot be entirely excluded. Future research, including studies with single-case experimental designs and randomized controlled trials, is necessary to rigorously evaluate the efficacy of MT for PPPD and address these limitations.

## Conclusions

MT was implemented for a patient with PPPD refractory to conventional treatments, leading to significant improvements in dizziness symptoms and QoL. A key contribution of this report is its presentation of a detailed application of MT for PPPD. To our knowledge, this is the first such case reported in English, suggesting MT's potential as a promising therapeutic alternative. The findings from this case suggest that MT's unique approach may be effective for PPPD via mechanisms different from those of other psychotherapies. Further systematic research is warranted to verify the effectiveness of MT for this condition and to explore its broader applicability.
